# Geographical disparities in obesity prevalence: small-area analysis of the Chilean National Health Surveys

**DOI:** 10.1186/s12889-022-13841-2

**Published:** 2022-07-29

**Authors:** Alejandro Sepúlveda-Peñaloza, Francisco Cumsille, Marcela Garrido, Patricia Matus, Germán Vera-Concha, Cinthya Urquidi

**Affiliations:** 1 Department of Epidemiology and Health Studies, Universidad de Los Andes, San Carlos de Apoquindo 2200, Las Condes, 7620001 Santiago, Chile; 2 Independent Consultant, Santiago, Chile

**Keywords:** Obesity, Small-area analysis, Geographic variation, Low-middle income households

## Abstract

**Background:**

Previous representative health surveys conducted in Chile evidenced a high obesity prevalence rate among adults, especially in female and urban areas. Nevertheless, these have limited utility for targeted interventions and local source allocation for prevention. This study analyzes the increments in obesity prevalence rates in populations ≥15 years of age and the geographic variation at the regional level. We also assessed whether the obesity rates have different patterns on a smaller geographic level than national and regional ones.

**Methods:**

This ecological study analyzed data from two representative national samples of adolescents and adults ≥15 years old, who participated in the last Chilean health surveys, 2009 (*n* = 5412) and 2016 (*n* = 6233). Obesity (body mass index≥30 kg/m^2^) rates were calculated on the national, regional, and Health service (HS) levels, being HS the smallest unit of analysis available. Obesity rates and relative increase to early identify target populations and geographic areas, with 95% confidence intervals (95% CI), were calculated using the sampling design of the national surveys, at the national and regional level, and by gender, age groups, and socioeconomic status. The Fay-Herriot (FH) models, using auxiliary data, were fitted for obesity rate estimates at the HS level.

**Results:**

The relative increase in obesity rate was 37.1% (95%CI 23.3–52.9) at the national level, with a heterogeneous geographic distribution at the regional one. Southern regions had the highest obesity rates in both surveys (Aysén: 35.2, 95%CI 26.9–43.5 in 2009, 44.3 95%CI 37–51.7 in 2016), but higher increases were predominantly in the northern and central areas of the country (relative increase 91.1 95%CI 39.6–110.1 in Valparaiso and 81.6 95%CI 14.4–196.2 in Tarapacá). Obesity rates were higher in females, older age, and lower socioeconomic groups; nevertheless, relative increases were higher in the opposite ones. The FH estimates showed an obesity rates variation at the HS level, where higher rates tend to converge to specific HS areas of each region.

**Conclusion:**

Obesity rates and relative increase are diverse across subnational levels and substantially differ from the national estimates, highlighting a pattern that converges to areas with low-middle income households. Our results emphasize geographical disparities in obesity prevalence among adults and adolescents.

**Supplementary Information:**

The online version contains supplementary material available at 10.1186/s12889-022-13841-2.

## Introduction

Halting the increase in obesity prevalence is an ever-pressing issue for public health policymakers. The worldwide rate of obesity has trebled over the past four decades, reaching 650 million (13%) and 1.9 billion (39%) obese and overweight adults in 2016, accounting for more deaths than undernutrition. This problem is more challenging in middle-low income countries due to the fact that the rapid upsurge of obesity coexists with undernutrition, while resources to deal with both are scarce [1, 2].

National Health Surveys (NHS) and complex surveillance systems are the usual frameworks to quantify the burden of diseases for planning interventions and resource allocation. Nevertheless, surveillance systems are seldom available in lower-middle-income countries. Moreover, the NHS is not updated as fast as needed and only represents large geographical areas (e.g., national and urban-rural levels). On the other hand, national-level or large geographical area estimates do not account for the disease variability in smaller geographical areas within a country, potentially missing information and target populations. For example, emerging evidence reported substantial geographical disparities in childhood mortality, cardiovascular and suicide rates [3–5], infectious diseases [6–8], and healthcare costs [9, 10] at subnational levels, highlighting the importance of geographic variation and the fast-growing demand for reliable small-area estimates. Concerning obesity, some studies based on NHS and surveillance records found a substantial difference in the small-area pattern of obesity rates among adults in high-income countries [11–16] and in children [17–19]. Since these studies cross-sectionally estimate obesity rates in smaller areas [20, 21], there is a gap of knowledge about the increase of the obesity rate among adults, especially in a setting where low-middle income households persist.

Chile can be a benchmark for anticipating the course and prevention policies of obesity in medium-low income settings due to the country experienced the fastest nutritional transition during the last four decades, tracking from high rates of undernutrition in the 1960s to high rates of obesity in the 2000s [22]. According to the World Bank, Chile has experienced the fastest growing economy in Latin America in recent decades; nevertheless, it has substantial socioeconomic variability. Thus, low-middle settings at subnational levels or lower geographic administrative divisions persist [23]. The last Chilean NHS reported that the obesity prevalence was 31% in 2016, affecting predominantly women and urban areas, with no examination of regional variation of the increase of obesity rates or the potential influence of smaller geographic areas.

The main objective of this study was to analyze the geographic variation of the increase of the obesity prevalence rates among adults and adolescents (≥ 15 years) at the national and regional levels, which are appropriate and valid for inference, comparing data from two representative NHS from Chile. Secondly, we assessed the geographic variation of the obesity rate in a smaller geographical unit related to health care services distribution, using small-area estimations (SAE).

## Method

### Design and data

We did a secondary analysis study based on the Chilean National Health Surveys (NHS) dataset. These datasets are held by the Subsecretaria de Salud Pública del Ministerio de Salud de Chile and are anonymized and freely available by request at https://www.portaltransparencia.cl/. Consequently, this study did not require ethical approval, consent to participate, and other administrative permission. All methods were carried out in accordance Declaration of Helsinki and Chilean regulations.

Chile is geographically divided into three administrative levels: regions (15 regions until 2017), provinces (56 provinces), and communes (346). On the other hand, Chile conducted three National Health Surveys (NHS). The last two NHS (NHS-2009 and NHS-2016) were considered for our analysis because sampling designs are similar; nevertheless, there are some differences in measured health conditions or risk factors. NSH-2009 and NSH-2016 are probabilistic samples of the general population aged 15 and over, with national and regional representativeness. The NHS’s four-stage sampling design includes a random sampling of communes, census zones, households, and individuals stratified by gender and urban-rural areas.

NHS-2009 was carried out between October 2009 and September 2010 and covered thirteen health conditions and other selected risk factors (alcohol, tobacco, food and salt consumption, passive exposure to environmental tobacco smoke). The participation rate was 85%; 5412 subjects completed the questionnaires and had clinical examinations, including laboratory tests and anthropometric measurements. The NHS-2016, developed between August 2016 to March 2017, extended its measures to 60 health conditions, including risk factors and population health determinants. The participation rate was 90%, 6233 subjects completed the health questionnaires, and 5220 had clinical and laboratory tests and anthropometry. In both surveys, health questionnaires, clinical examinations, and measured height (in centimeters) and weight (in kilograms) data were completed by trained nurses and study staff visiting each selected household and following standardized procedures and validated instruments/equipment. The variables analyzed were weight, height, gender, age, education level, the region of residence, and the individual’s health care system, including the HS records explained later. Only complete data from both surveys were included in our analysis.

### Definition of obesity and subnational geographic levels

We used the Word Health Organization’s criteria to define adult obesity: body mass index (BMI) over or equal to 30. We calculated BMI as weight in kilograms divided by the square of the height in meters.

Chilean NHS has national, regional, gender, and urban-rural areas representativeness but does not collect representative data at the country’s second and third geographic administrative divisions (provinces and commune). Each participant’s commune or province of residence is not wholly and reliably registered either. Instead, we constructed a smaller geographical area using ancillary data recorded on both NHS, indirectly related to the commune or province, and the HS. The health system in Chile is based on 29 sub-regional geographical HS, which aim to manage and develop a public healthcare network under a defined jurisdiction, being different between them in terms of geographical area extension, population density, educational levels, and family income. In this way, we analyzed obesity prevalences rates at three geographical levels: national, regional, and HS areas, being the HS the smallest unit of analysis available.

By the time of NHS (2016–2017), Chile was administratively divided into 15 regions from north to south. Regions are grouped only for the interpretative purpose by the following macro zones: North zone, Central zone, Central-south, South, and Austral zone.

### Data processing

All analyses accounted for the complex sampling design to produce population-based weighted nationally representativeness according to Chilean NSH analytic guidelines.

At the national and regional levels, we calculated obesity prevalence rates (obesity rate from now on) with 95% confidence intervals (95% CI) using the sampling expansion factors (Chilean NSH guidelines); we named this the traditional approach (TA). To estimate the absolute (difference between obesity rate in NHS-2016 and NHS-2009) and relative increase rates (ratio between difference and obesity rate in NHS-2009) and their 95% CI, we fitted linear regression and log-linear models; both models consider the probabilities of inclusion of each survey. These rates were also calculated for the following subpopulation: gender (male, female), age groups (15-24y, 25-44y, 45-64y 65y or more), and education level as a socioeconomic status proxy variable (low, medium, high). Due to Chile already having elevated obesity rates (NSH-2016), we emphasize the relative increase indicator, rather than the absolute one, to identify target areas or subgroups for early prevention since this indicator depends on its baseline value.

For SAE at the HS level, we used Fay-Herriot (FH) models for the obesity rates estimations. We preferred FH to other emerging SAE models because it is a well-known lineal-mixed-model approach to fill the data gap over small geographic areas. It accounts for too small sample sizes and provides accurate direct estimates [24]. Two types of models were fitted:

FH model:

$${\hat{\delta}}_d^{DIR}={\delta}_d+{e}_d,{e}_d\sim N\left(0,{\psi}_d\right),\mathrm{and}\ d=1,\dots, D.$$

$${\delta}_d={x}_d^T\beta +{u}_d,\mathrm{where}\ {u}_d\sim N\left(0,A\right),\mathrm{and}\ d=1,\dots, D$$

Where *δ*_*d*_ is the parameter to be estimated in the area *d* (*d* = 1, …, *D*). ${\hat{\delta}}_d^{DIR}$ is the unbiased directed estimator (DIR) obtained with the sample design. *ψ*_*d*_ is the sample variance of the direct estimator in each area. ${x}_d^T$ are the auxiliary linear predictors related to variables of interest, and A is a matrix D × D, where D is the geographic unit or area of analysis.

SFH model is the spatial Fay-Herriot model (SFH) to reduce estimation variance at under-sampled areas and spatial autocorrelation due to neighborhood distance, which assumes that *u* = (*u*_1_, …, *u*_*D*_) related to the areas that follow a first-order autoregressive process SAR [1]. That is:

$$u={\rho}_1 Wu+\epsilon, \mathrm{where}\ \epsilon \sim N\left({0}_D,{\sigma}_1^2{I}_D\right).$$

Where 0_*D*_ is a vector of zeros, and *I*_*D*_ the identity matrix. *W* is the proximity matrix D × D obtained by a row standardization of an initial matrix with zeros and ones, where the number 1 indicates if the areas are neighbors. *ρ*_1_ is a scalar parameter.

The area-level auxiliary data and covariates used for SAE were children’s obesity/overweight and mortality rates at the commune level, population size, and the number of medical establishments at the HS level. These data are public and accessible in Chile [25].

Direct (DIR), FH, and SFH estimates with standard errors (SE) were reported. DIR is calculated using data exclusively from the NHS and is similar to the obesity rate with TA. We fitted log-linear models and the FH and SFH modes for the relative increase in HS areas, and later FH and SFH models were fitted.

Graphs and maps to show geographic variation were also constructed for results visualization. All the analyses were conducted in R version 4.1.1 [26]. Figures and maps are produced using R packages *ggplot* (version 3.3.5), *ggrepel* (version 0.9.1 [23], *rgdal* (version 1.5–23), *rgeos* (version 0.5–5), and *chilemapas* (version 0.2). Obesity rates were calculated using the package *survey* (version 4.1–1), and FH models were estimated using package *SAE* version 1.3.

## Results

A total of 4884 (90.2%) and 5220 (83.7%) complete registers of NSH-2009 and NSH-2016, respectively, were analyzed in this study. Sample size distribution by the regional level and regions aggregated by macro zones are displayed in Table 1. Chile has 15 regions with 29 HS imbalanced distributed across each region. For instance, the Valparaiso, Metropolitan, Biobío, La Araucanía, and Los Lagos regions have from 2 to 6 HS areas; in contrast, there is just one HS for the rest of the regions (Table 1).

**Table 1:** Health service areas and sample size distribution by Chilean regions and macro zones

Macro zone	Regions^**a**^	NHS-2009	NHS-2016	HS
North	Arica y Parinacota	290	303	1. Arica
	Tarapacá	279	285	2. Iquique
	Antofagasta	290	272	3. Antofagasta
	Atacama	291	281	4. Atacama
Central	Coquimbo	287	293	5. Coquimbo
	Valparaíso	316	580	6. Valparaíso San Antonio 7. Viña del Mar Quillota 8. Aconcagua
	Metropolitana	817	832	1. M. Norte 2. M. Occidente 3. M. Central 4. M. Oriente 5. M. Sur 6. M. Suroriente
Central-south	L. Bdo. O’Higgins	309	292	1. O’Higgins
	Maule	329	340	2. Del Maule
	Biobío	263	601	3. Ñuble 4. Concepción 5. Talcahuano 6. Biobío 7. Arauco
South	La Araucanía	291	273	1. Araucanía norte 2. Araucanía sur
	Los Ríos	286	289	1. Valdivia
	Los Lagos	288	297	1. Osorno 2. Reloncavi 3. Chiloe
Austral	Aysén	252	307	1. Aysén
	Magallanes	297	275	2. Magallanes

*NHS* National Health Survey, *HS* Health service

^a^ Regions are ordered according to their geographic location, from north to south

Table 2 shows the sample size distribution by the subpopulations of the study. In NSH-2009, 59.7% of the sample were female, 24.7% older than 65 years, and 26.6% low socioeconomic status. While 63.4% were female, 24.7% older than 65 years, and 22.3% had low socioeconomic status in the NSH-2016 (Table 2).

**Table 2:** Sample size distribution by subpopulations in NHS-2009 and NHS-2016, Chile

	NHS-2009	NHS-2016
Gender		
Male	1966 (40.3)	2019 (36.6)
Female	2918 (59.7)	3501 (63.4)
Age groups		
15 to 24 y	727 (14.9)	729 (13.2)
25 to 44 y	1613 (33.0)	1571 (28.5)
45 to 64 y	1611 (33.0)	1857 (33.6)
65 y or more	933 (19.1)	1363 (24.7)
Socioeconomic status		
Low	1297 (26.6)	1229 (22.3)
Medium	2658 (54.4)	2948 (53.4)
High	912 (18.7)	1196 (21.7)
Total	4884	5520

*NHS* National Health Survey, *HS* Health service

### Obesity rates, absolute and relative increase at regional levels and by subpopulations

**Fig. 1:**
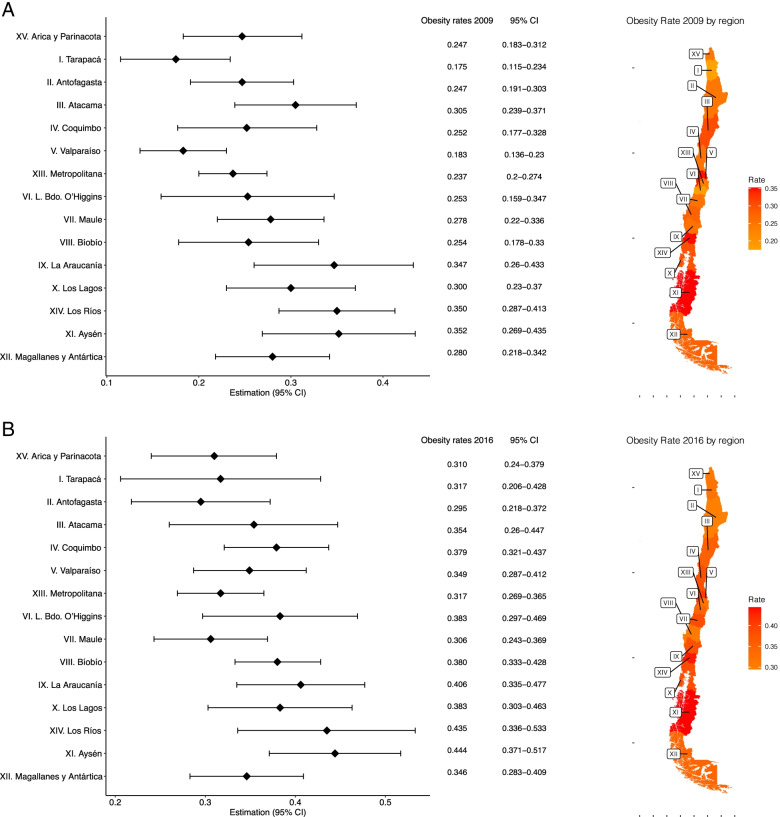
Distribution of the obesity rates in National Health Survey 2009 (A), 2016 (B) by regions, Chile. Obesity rates and 95%CI are illustrated as proportions. NSH: National Health Survey. 95%CI: 95% Confidence Intervals. *Regions are geographically displayed from north to south. The vertical dashed line represents the obesity rate at the national level

At the national level, the obesity rates in the NHS-2009 and NSH-2016 were 25.1% (95%CI 23.0–27.2) and 34.4% (95%CI 32.1–36.8), respectively, representing a significant relative increase of 37.1% (95%CI 23.3–52.9). Figure 1 is the map of Chile where regions are geographically displayed from north to south and obesity rates with 95%CI, showing the heterogeneous geographic distribution of obesity rates in both surveys regarding the regional level. The highest obesity rates were concentrated in the country’s southern regions in both surveys; meanwhile, in regions from the north and center zones of the country, obesity rates oscillate around or lower than the national average in both surveys (Fig. 1A and B). Table 4 of supplemental material contains the obesity rates, absolute and relative increase with 95%CI at national and regional levels. (Supplement, Table 4).

**Fig. 2:**
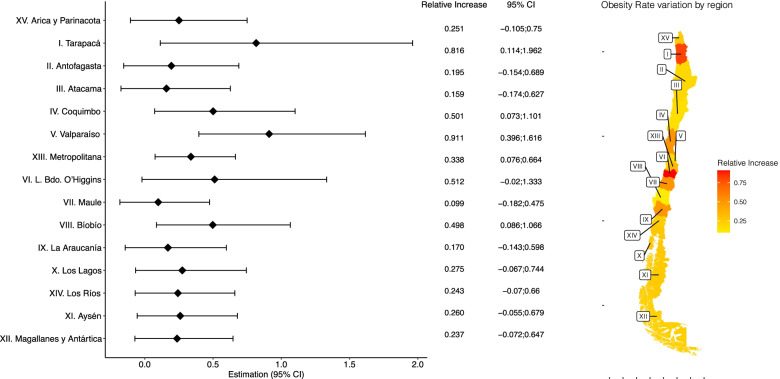
Obesity prevalence relative increase rate from NHS-2009 to NHS-16 at the regional level, Chile. Obesity relative increase rates and 95%CI are illustrated as proportions. NSH: National Health Survey. 95%CI: 95% Confidence Intervals. *Regions are geographically displayed from north to south. The vertical dashed line represents the obesity rate at the national level

Figure 2 illustrates the obesity relative increase rates by region, meaning the reference line the no increments. There is also a geographical variation in obesity relative increase rates; however, this is predominantly in the northern and central zones of the country (Fig. 2). Highest obesity relative increase was in Tarapacá (81.6, 95%CI 11.4–196.2) and Valparaíso (91.1, 95%CI 39.6–116.6). Moreover, three regions from the central and central-south macron zones had a significant relative increase: Coquimbo (50.1, 95%CI 7.3–110.1), Biobío (49.8, 95%CI 8.6–106.6), and Metropolitan region (33.8, 95%CI 7.6–66.4) (Fig. 2). These results and the absolute increase are detailed in the supplementary material (supplement, Table 4).

Regarding subpopulations, the female obesity rate was higher than males in both surveys; nevertheless, obesity relative increase in males was higher than in females (57.0, 95%CI 31.5–87.4 vs. 25.7, 95%CI 10.7–42.7). Similarly, obesity rates were higher in older age groups, but relative increases were higher in the younger ones (62.4, 95%CI 11.9–135.6; 53.2 95%CI 26.9–85.0 vs. 19.1, 95%CI 2.3–38.6; 14.9, 95%CI 7.4–42.7). Educational level is used as a proxy for the socioeconomic status in Chilean NSH. In this sense, low and medium socioeconomic subgroups have higher obesity rates than their counterparts, although a high relative increase was in the high-socioeconomic group (58.7, 95%CI 19.4–110.9 vs. 32.8, 95%CI 11.6–57.9 and 35.1, 95%CI 17.7–55.1) (Table 3).

**Table 3:** Obesity indicators by subpopulations in Chile

	Prevalence (95%CI) NHS-2009	Prevalence (95%CI) NHS-2016	Absolute increase	Relative increase %
Gender				
Male	19.3 (16.5–22)	30.3 (27.1–33.5)	11.0 (6.8–15.2)	57.0 (31.5–87.4)
Female	30.6 (27.6–33.5)	38.4 (35.3–41.6)	7.9 (3.5–12.2)	25.7 (10.7–42.7)
Age groups				
15 to 24 y	11.0 (7.6–14.3)	17.8 (14.0–21.6)	6.8 (1.8–11.9)	62.4 (11.9–135.6)
25 to 44 y	23.6 (20.0–27.1)	36.1 (31.9–40.3)	12.5 (7.1–18.0)	53.2 (26.9–85.0)
45 to 64 y	35.4 (31.4–39.5)	42.2 (38.0–46.4)	6.8 (0.9–12.6)	19.1 (2.3–38.6)
65 y or more	31.0 (25.6–36.4)	35.6 (31.1–40.1)	4.6 (2.4–11.7)	14.9 (7.4–42.7)
Socioeconomic status				
Low	35.1 (30.6–39.6)	46.6 (41.1–52.1)	11.5 (4.4–18.6)	32.8 (11.6–57.9)
Medium	24.7 (22.2–27.3)	33.4 (30.5–36.3)	8.7 (4.7–12.6)	35.1 (17.7–55.1)
High	18.5 (14.1–22.9)	29.4 (24.8–34.1)	10.9 (4.5–17.3.2)	58.7 (19.4–110.9)

95% CI: 95% Confidence Interval. NHS: National Health Survey

^a^ Calculated according to the Chilean NHS traditional approach

^b^ Difference between obesity rates in NHS-2016 and NHS-2009

^c^ The ratio between the difference in the obesity rate in NHS-2009) × 100

**Fig. 3:**
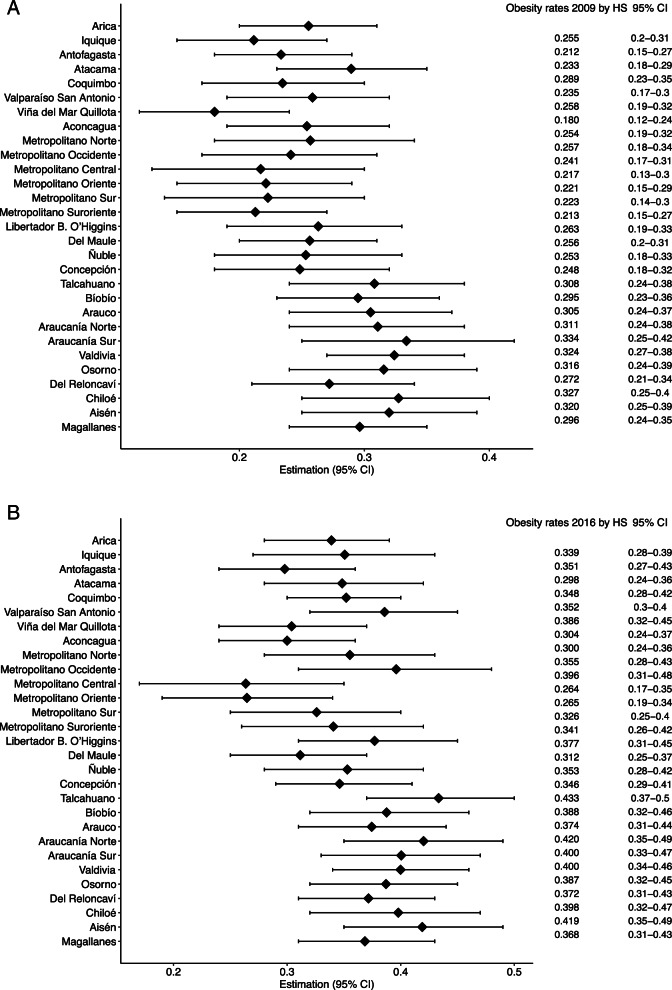
Spatial Fay-Herrot obesity rates estimates by health services areas, Chile. NSH: National Health Survey. 95%CI: 95% Confidence Intervals. HS: Health service. *HS are geographically displayed from north to south. The vertical dashed line represents the obesity rate at the national level

Figure 3 shows the SFH model’s obesity rate estimates and 95%IC for the 29 HS areas of Chile in NHS-2009 and NSH-2016, where HS areas are displayed from north to south. A geographic variation of obesity rates at the HS level was observed in both surveys. HS areas with an obesity rate over the national average tend to converge to the country’s southern zone in NHS-2009 and NHS-2016. Figure 3 also highlights how certain HS areas in the north and central Chilean macro zones also had high obesity rates according to NHS-2016.

Figure 5 (supplementary material) shows that FH and SFH obesity rate estimates tend to be similar to the unbiased direct estimator (Direct) in HS areas but with less variability in their standard error. Table 5 (supplementary material) shows the obesity rates estimates from the Direct Fay-Herriot models and sample size at the HS level only in regions with two or more HS areas to illustrate the variability of obesity rates within their respective regions. Additionally, FH and SFH estimates tend to be similar.

Araucanía and Los Lagos Regions, from the south zone, had the highest obesity rates in both surveys and were similar in all their HS areas (supplement, Table 5). The obesity rate in Valparaiso Region (central zone) 2 of its 3 HS areas already had a prevalence similar to the national one (25.8% in San Antonio and 25.4% in Viña del Mar, 25.1% the national rate), even higher in the San Antonio HS area in NSH-2016 (38.6% vs. 34.9% national rate). The Metropolitan region is one of the most populated in Chile. The obesity rates at HS areas in this region tended to be homogenous in NSH-2009; however, it is observable a major geographic variation across its HS areas in NSH-2016, where the highest obesity rates concentrate in 2 of its 6 HS areas (39.6% in M. Occidente and 35.5% in M. Norte). This variability at the HS level was also observable in the Biobío region from the central-south zone (Supplement, Table 5).

**Fig. 4:**
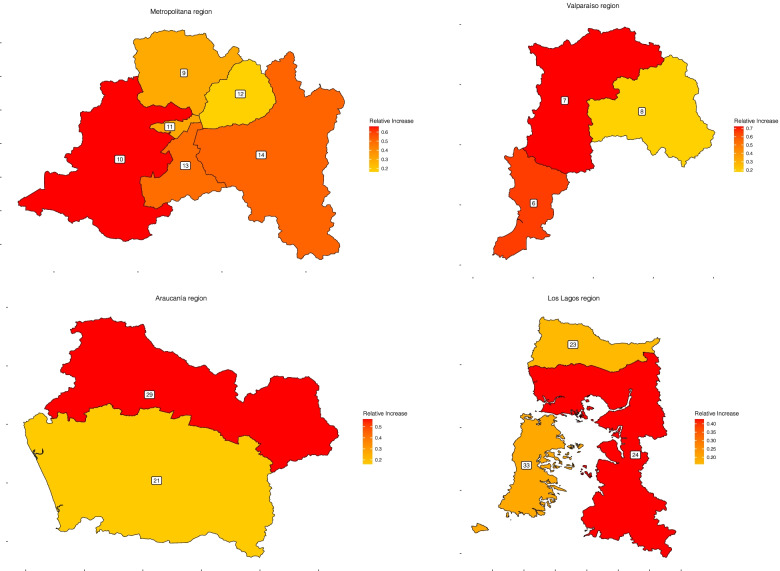
Relative increase rate in obesity in regions with two or more HS areas, Chile. HS: Health service. HS in Mtropolitana Region: 9. M. Norte. 10. M. Occidente. 11. M.Central. 12. M. Oriente. 13. M. Sur. 14. M. Suroriente. Valparaiso Region: 6. Valparaíso San Antonio. 7. Viña del Mar Quillota. 8. Aconcagu Araucania Region: 22. Araucanía sur. 23. Araucanía norte. Los Lagos Region:. 25. Osorno. 26. Reloncaví. 27. Chiloé

Regarding obesity relative increase at the HS level, there is also a variation between HS areas belonging to the same region; for example, in the Valparaiso region, a region with the highest obesity prevalence and relative increase rates, this is concentrated in certain HS areas (Fig. 4).

## Discussion

To our knowledge, this is the first study that reported the geographic variation of obesity rate increments and obesity rates at smaller geographic areas in Chile. The main purpose of studying geographic variation in disease rates is to provide health management information and formulate hypotheses about the environmental determinants of disease. The “hot spot” can also be identified as a high concentration of cases in a particular area. Subsequently, the correlation between geographic disease variation and concomitant variation in the degree of exposure to the environment or lifestyle can be addressed [13, 24]. Identifying small areas of highest prevalence allows to address the health consequences of obesity and provide specific resources to provide an early diagnosis and timely treatment. Moreover, identifying environmental drivers will promote better urban planning [27, 28].

Chile presents a great regional variation in socio-economic development and disparities in health indicators [23]. Consequently, our study’s results show that the prevalence of obesity and its increments are very heterogeneous in the territory, even within regions. Disclosing that the “hot spots” are different between subnational levels and smaller areas, represented by regions and the HS, respectively, and suggesting geographic disparities in obesity. Scrutinizing our results, HS areas with higher obesity prevalence coincide with less developed communes in Chile, according to the Community Development Indicator (CDI) [23], even in regions with better socioeconomic development. However, this seems to be different in the relative increments.

The Metropolitan Region (the region with the most population density), as an example, has the highest CDI (0.50), and its obesity rate is near to the national average (31.7%, NHS-2016). Nevertheless, HS areas belonging to less developed communes showed higher obesity rates (M. Occidente: 40%, M. Norte 35%), contrary to those with higher CDI (M. Oriente 26%). On the other hand, other HS areas have the largest relative increases (M.Central and M. Suroriente). Even though there is a knowledge gap regarding factors that could explain the observed differences, further hypotheses to be tested are the influence of socioeconomics or educational factors, and other environmental drivers of obesity. Literature also suggests access to green areas that encourage physical activity, safe public transport planning and promote joint mobility with bicycles and walks, barriers to the consumption of junk food, etc.

In addition, our findings based on the relative obesity increases indicators make visibly hidden “hot spots” susceptible to earlier preventive interventions. Males, the youngest age group, highest socioeconomic status, and regions from the central and norths zone of the country are becoming the targeted groups of concern. High prevalence and the emerging relative obesity increases in other subpopulations also lead to reflection on the public policy measures taken in the country to solve or confront the problem. The establishment of laws (such as nutrition labeling) as the only preventable intervention has been insufficient to curb obesity. The above reinforces the importance of examining prevalence at sub-national labels and the need to adjust preventive measures to the local reality to face the determinants of this problem. These can be studied using modern approaches such as big data, artificial intelligence, geo-analysis, agent-based models, and public health dashboards [29].

Strengths of our study include the large sample size, which represents adolescents and adults 15 years of age and older, and the standardized weight and height measurements conducted by trained personnel in both surveys, which gives reliable indicators for comparison. Traditional and well-known modeling for SAE allowed us to estimate obesity prevalence in smaller areas, where sample sizes are too small or insufficient. Although the above could explain the high prevalence due to high variability, evidence supports the reliability of different SAE approaches; moreover, our interim analysis reinforces the robustness of our estimates (Supplement, Fig. 5). Still, local surveillance data for direct estimation would be preferable, and local government should consider resources to better track obesity and other obesity-related chronic health conditions at this level.

The principal limitation of our study is that we have no complete data at the province or commune level. Instead, we use HS data as a proxy variable and the education level as a proxy socioeconomic status. HS data may misrepresent communes, especially in regions with less health service access or rural areas. Nevertheless, this is the first study exploring the utility of this data and brightening to light important geographic variations in obesity prevalence among adults and adolescents. In addition, due to the potential for bias, ecological studies should only be considered the first step in investigating the potential associations between environmental agents and disease geography differences [30].

In conclusion, this study determined that the obesity rates and relative increase at subnational levels substantially differ from the national estimates, highlighting geographical disparities in obesity prevalence among adults and adolescents. These findings suggest that stakeholders should stake prevention programs and resource investment locally and evaluate determinants of these disparities and their impact on other diseases.

## Supplementary Information

**Additional file 1.**

## Data Availability

The data supporting this study’s findings are held by the Subsecretaria de Salud Pública del Ministerio de Salud de Chile and are publicly available at request from https://www.portaltransparencia.cl/. However, data are available from the authors upon reasonable request and with the permission of Alejandro Sepulveda, the first author of the study.

## Abbreviations

NHS: National Health Survey
HS: Health Service
BMI: Body Mass Index
TA: Traditional Approach
FH: Fay-Herriot estimates
SFH: Spatial Fay-Herriot estimates
95%CI: 95% Confidence Interval
SE: Standard Errors
