# Supplementary material for: Geographical disparities in obesity prevalence: small-area analysis of the Chilean National Health Surveys
Source: BMC Public Health. 2022 Jul 29;22:1443. doi: 10.1186/s12889-022-13841-2 (PMC9335969; doi:10.1186/s12889-022-13841-2)
Supplement: Supplementary file 1 — Additional file 1. [file 12889_2022_13841_MOESM1_ESM.doc]

**Supplementary**

**Table 4.** **Obesity indicators (%) by national and regional levels in Chile.**

|  | **NHS-2009^a^**  **(95%CI)** | **NHS-2016^a^**  **(95%CI)** | **Absolute increase^b^**  **(95%CI)** | **Relative increase^c^**  **(95%CI)** |
| --- | --- | --- | --- | --- |
| National level | 25.1 (23.0-27.2) | 34.4 (32.1-36.8) | 9.3 (6.2-12.5) | 37.1 (23.3-52.9) |
| Regional level |  |  |  |  |
| Arica y Parinacota | 24.7 (18.3-31.2) | 31.0 (24.0-37.9) | 6.3 (5.7-6.7) | 25.1 (21.5-31.1) |
| Tarapacá | 17.5 (11.4-23.4) | 31.7 (20.6-42.8) | 14.3 (1.7-26.9) | 81.6 (11.4-196.2) |
| Antofagasta | 24.7 (19.1-30.3) | 29.5 (21.8-37.2) | 4.8 (4.7-14.3) | 19.5 (-15.4-68.9) |
| Atacama | 30.5 (23.9-37.1) | 35.4 (26.0-44.7) | 4.9 (6.5-16.2) | 16.2 (-17.4-62.7) |
| Coquimbo | 25.3 (17.7-32.8) | 37.9 (32.1-43.7) | 12.6 (3.1-22.2) | 50.1 (7.3-110.1) |
| Valparaíso | 18.3 (13.6-23.0) | 34.9 (28.7-41.2) | 16.7 (8.8-24.5.2) | 91.1 (39.6-116.6) |
| Metropolitana | 23.7 (18.0-26.4) | 31.7 (26.9-36.5) | 8.0 (1.9-14.1) | 33.8 (7.6-66.4) |
| L. Bdo. O´Higgins | 25.3 (15.9-34.7) | 38.3 (29.7-46.9) | 13.0 (0.2-25.7) | 51.2 (-2.0-133.3) |
| Maule | 27.8 (22.0-33.6) | 30.6 (24.3-36.9) | 2.7 (5.9-11.4) | 9.9 (-18.2-47.5) |
| Biobío | 25.4 (17.8-33.0) | 38.0 (33.3-42.8) | 12.6 (3.8-21.5) | 49.8 (8.6-106.6) |
| La Araucanía | 34.7 (26.0-43.3) | 40.6 (33.5-47.7) | 5.9 (5.6-17.4) | 17.0 (-14.3-59.8) |
| Los Ríos | 35.0 (28.7-41.3) | 43.5 (33.6-53.3) | 8.5 (3.2-20.2) | 24.3 (-7.0-66.0) |
| Los Lagos | 30.0 (23.0-37.0) | 38.3 (30.3-46.3) | 8.3 (2.4-18.9) | 27.5 (-6.7-74.4) |
| Aysén | 35.2 (26.9-43.5) | 44.3 (37.1-51.7) | 9.1 (1.9-20.2) | 26.0 (-5.5-67.9) |
| Magallanes | 28.0 (21.8-34.2) | 34.6 (28.3-40.9) | 6.6 (2.2-15.5) | 23.7 (-7.2-64.7) |

95% CI: 95% Confidence Interval. NHS: National Health Survey

a Calculated according to the Chilean NHS traditional approach.

b Obesity rates in NHS-2016 minus obesity rate in NHS-2009

c Difference between obesity rates (NHS-2016 and NHS-2009), divided by rates in NHS-2009 × 100

**Table 5.** **Obesity rates estimates from the unbiased direct estimator, Fay-Herriot models at the HS level**

| **Region** | **HS**  **area** | **NSH-2009** | | | | **NSH-2016** | | | |
| --- | --- | --- | --- | --- | --- | --- | --- | --- | --- |
|  |  | **n** | **DIR** | **FH** | **SFH** | **n** | **DIR** | **FH** | **SFH** |
| Valparaíso | Valparaíso San Antonio | 88 | 21.3 (4.7) | 26.7 (3.3) | 25.8 (3.3) | 160 | 46.4 (7.2) | 40.5 (3.9) | 38.6 (3.5) |
|  | Viña del Mar Quillota | 177 | 15.5 (3.2) | 18.1 (2.8) | 18.0 (2.9) | 317 | 32.2 (3.7) | 31.2 (3.1) | 30.4 (3.2) |
|  | Aconcagua | 51 | 24.6 (5.2) | 25.3 (3.5) | 25.4 (3.5) | 103 | 24.2 (4.2) | 31.0 (3.3) | 30.0 (3.2) |
| Metropolitana | M. Norte | 91 | 34.7 (6.9) | 26.1 (3.5) | 25.7 (3.5) | 116 | 35.1 (7.0) | 36.4 (4.1) | 35.5 (4.0) |
|  | M. Occidente | 131 | 24.3 (4.1) | 23.9 (3.5) | 24.1 (3.5) | 147 | 43.6 (6.1) | 39.1 (4.5) | 39.6 (4.3) |
|  | M. Central | 116 | 22.0 (4.8) | 18.6 (4.6) | 21.7 (4.3) | 137 | 28.4 (5.1) | 24.3 (5.0) | 26.4 (4.6) |
|  | M. Oriente | 164 | 22.2 (4.7) | 22.3 (4.2) | 22.1 (3.7) | 116 | 21.2 (6.2) | 25.3 (5.0) | 26.5 (4.0) |
|  | M. Sur | 133 | 22.0 (4.6) | 23.0 (4.0) | 22.3 (4.1) | 142 | 32.3 (4.0) | 33.2 (3.7) | 32.6 (3.7) |
|  | M. Suroriente | 182 | 20.5 (3.3) | 19.4 (3.2) | 21.3 (3.1) | 174 | 27.9 (5.7) | 31.6 (4.7) | 34.1 (4.3) |
| Biobío | Ñuble | 54 | 17.8 (8.4) | 25.9 (3.9) | 25.3 (3.8) | 123 | 36.8 (4.6) | 36.7 (3.5) | 35.3 (3.5) |
|  | Concepción | 61 | 16.8 (5.0) | 23.6 (3.8) | 24.8 (3.4) | 200 | 30.8 (3.7) | 34.2 (3.3) | 34.6 (3.1) |
|  | Talcahuano | 55 | 36.3 (7.6) | 29.3 (3.9) | 30.8 (3.6) | 131 | 47.3 (4.1) | 42.6 (3.3) | 43.3 (3.3) |
|  | Bíobío | 46 | 35.0 (8.5) | 30.4 (3.5) | 29.5 (3.5) | 108 | 43.1 (7.2) | 40.1 (3,7) | 38.8 (3.5) |
|  | Arauco | 47 | 34.7 (7.2) | 30.4 (3.6) | 30.5 (3.5) | 39 | 36.8 (6.6) | 39.2 (3.7) | 37.4 (3.5) |
| La Araucanía | Araucanía Sur | 236 | 35.7 (5.3) | 35.0 (4.2) | 33.4 (4.2) | 214 | 38.2 (3.9) | 40.4 (3.6) | 40.0 (3.5) |
|  | Araucanía Norte | 55 | 30.5 (5.8) | 31.6 (3.7) | 31.1 (3.4) | 59 | 54.7 (8.4) | 44.8 (4.2) | 42.0 (3.6) |
| Los Lagos | Osorno | 89 | 35.3 (6.5) | 31.8 (3.8) | 31.6 (3.7) | 76 | 39.3 (5.5) | 41.7 (3.9) | 38.7 (3.4) |
|  | Del Reloncaví | 131 | 24.0 (5.1) | 28.1 (3.2) | 27.2 (3.3) | 140 | 38.0 (5.0) | 37.8 (3.4) | 37.2 (3.2) |
|  | Chiloé | 68 | 35.7 (7.4) | 33.7 (3.7) | 32.7 (3.8) | 81 | 37.3 (10) | 41.2 (4.1) | 39.8 (3.9) |

% and Standard Error in (). NHS: National Health Survey. HS: Health Service.

DIR: unbiased direct estimator for obesity rate from Fay-Herriot models using a sample size of NSH.

FH. Fay-Herriot obesity rates estimates for small areas.

SFH. Spatial Fay-Herriot obesity rates estimates for small areas.

### Figure 5. Comparison of obesity rates from the unbiased direct estimate and small areas estimator, NSH-2016

1.
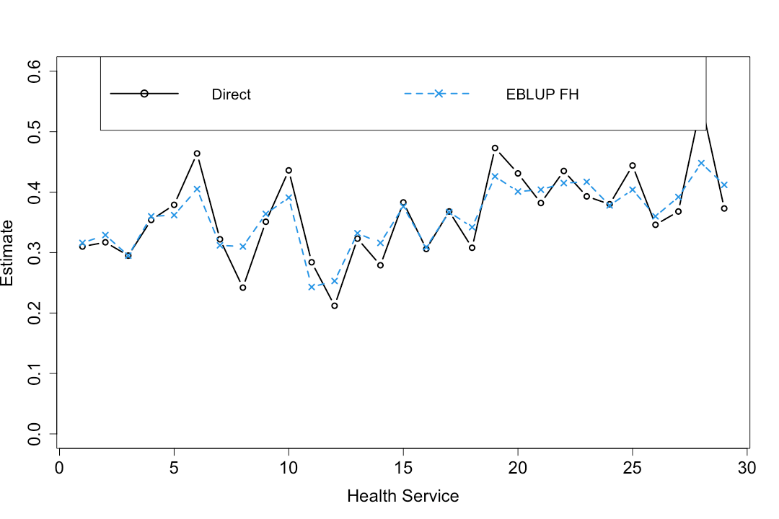
**Fay-Herriot model estimates**

**
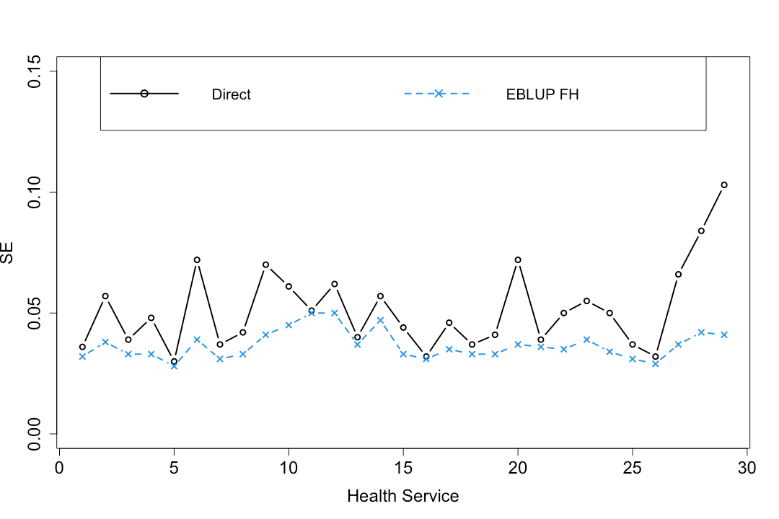
**

1.
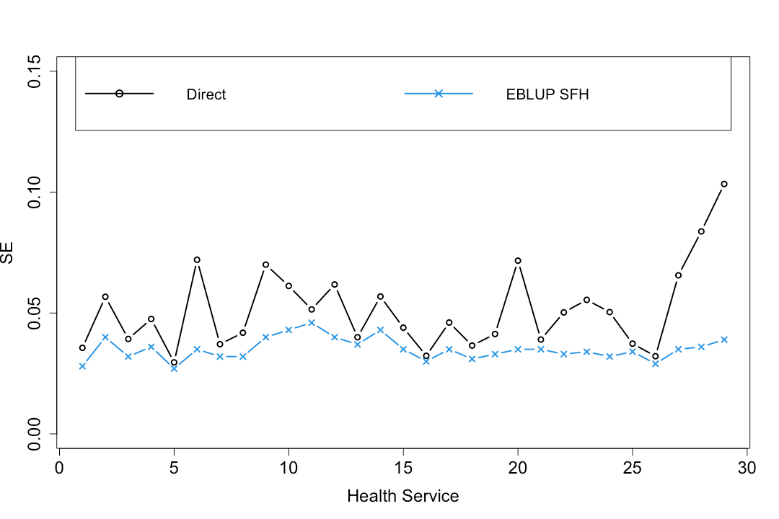
**Spatial Fay-Herriot model estimates**

**
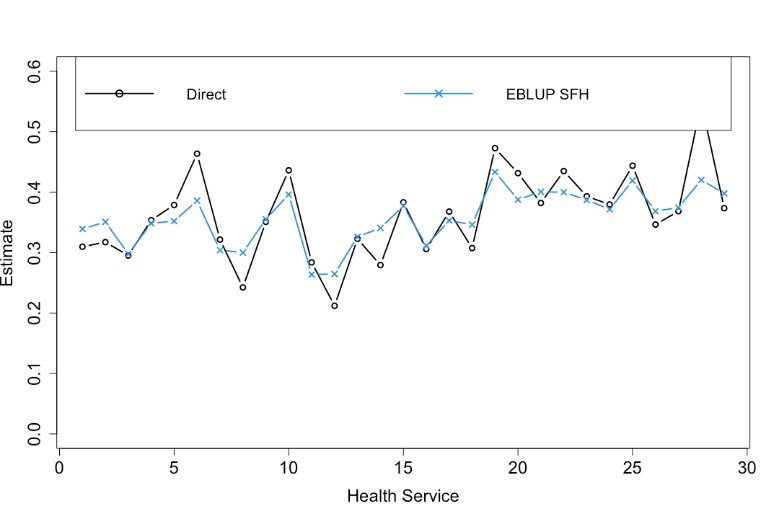
**

NHS: National Health Survey. HS: Health Service. EBLUP: Empirical Best Linear Unbiased Predictor.

Direct: unbiased direct estimator for obesity rate from Fay-Herriot models using a sample size of NSH.

FH. Fay-Herriot obesity rates estimates for small areas.

SFH. Spatial Fay-Herriot obesity rates estimates for small areas.

**Table 6. Obesity rates at the regional level with the traditional approach and small-area estimation**

|  | **NSH-2009** | | | **NSH-2016** | | |
| --- | --- | --- | --- | --- | --- | --- |
| **Regions** | **TA** | **FH** | **SFH** | **TA** | **FH** | **SFH** |
| XV Arica y Parinacota | 24.7 | 24.3 | 25.5 | 31.0 | 31.2 | 31.2 |
| I. Tarapacá | 17.5 | 19.9 | 19.7 | 31.7 | 33.6 | 33.6 |
| II. Antofagasta | 24.7 | 23.8 | 23.6 | 29.5 | 29.9 | 29.9 |
| III. Atacama | 30.5 | 29.6 | 28.5 | 35.4 | 37.1 | 37.1 |
| IV. Coquimbo | 25.3 | 25.9 | 26.5 | 37.9 | 37.2 | 37.1 |
| V. Valparaíso | 18.3 | 19.3 | 19.6 | 34.9 | 33.4 | 33.3 |
| XIII. Metropolitana | 23.7 | 23.5 | 23.4 | 31.7 | 31.7 | 31.7 |
| VI. O´Higgins | 25.3 | 28.8 | 28.4 | 38.3 | 40.3 | 40.6 |
| VII. Maule | 27.8 | 26.8 | 26.9 | 30.6 | 33.2 | 33.3 |
| VIII. Biobío | 25.4 | 26.2 | 25.8 | 38.0 | 36.6 | 36.4 |
| IX. La Araucanía | 34.7 | 34.5 | 36.2 | 40.6 | 40.7 | 40.8 |
| X. Los Lagos | 30.0 | 29.9 | 28.7 | 38.3 | 36.6 | 36.4 |
| XI. Aysén | 35.2 | 31.8 | 31.7 | 44.3 | 38.8 | 38.9 |
| XII. Magallanes | 28.0 | 28.5 | 28.2 | 34.6 | 37.3 | 37.2 |
| XIV. Los Ríos | 35.0 | 33.8 | 34.3 | 43.5 | 42.4 | 42.4 |

% and () Sttader Error.

NHS: National Health Survey

TA: Traditional Approach.

FH. Fay-Herriot obesity rates estimates for small areas.

SFH. Spatial Fay-Herriot obesity rates estimates for small areas.
